# The Prevalence and Overlaps of Temporomandibular Disorders in Patients with Myofascial Pain with Referral—A Pilot Study

**DOI:** 10.3390/ijerph18189842

**Published:** 2021-09-18

**Authors:** Joanna Kuć, Krzysztof Dariusz Szarejko, Maria Gołębiewska

**Affiliations:** 1 Department of Prosthodontics, Medical University of Bialystok, 24A M. Sklodowskiej-Curie St., 15-276 Bialystok, Poland; 2 Private Health Care, Physical Therapy and Rehabilitation, 79 Warsaw St., 15-201 Bialystok, Poland; biuro@rehabilitacja-lecznicza.pl; 3 Department of Dental Techniques, Medical University of Bialystok, 13 Washington St., 15-269 Bialystok, Poland; maria.golebiewska5@gmail.com

**Keywords:** joint vibration analysis, myofascial pain with referral, temporomandibular disorder, temporomandibular joint

## Abstract

The aim of the study was to evaluate the temporomandibular joint, the prevalence of single and multiple diagnosis and potential sided domination of temporomandibular dysfunction in patients with temporomandibular disorder—myofascial pain with referral. The study group enrolled 50 people—37 females and 13 males between 18 and 25 years old with an average age of 23.36 ± 2.14. The patients underwent joint vibration analysis. Sixty seven percent of all examined tem-poromandibular joints were classified as group I according to Mark Piper’s classification. Class IIIA appeared in 17% of joints. Eight percent of temporomandibular joints were classified as class IVA. There were no statistically significant differences in the prevalence of temporomandibular disorder with respect to gender (*p* = 0.838639). The relatively high prevalence of multiple diagnoses proved the overlapping nature of muscle and intraarticular disorders. Twenty eight percent of the subjects suffered from a combination of myofascial pain with referral and bilateral temporoman-dibular dysfunction. In 62% of the patients a lack of intraarticular disorders was reported. The suggestion that there exists sided domination in the occurrence of temporomandibular disorders has not been confirmed. Due to the small sample size, such differences cannot be excluded. Fur-ther research is needed.

## 1. Introduction

Temporomandibular disorders (TMDs) affect approximately 5% to 12% of the adult population [1]. In children, the prevalence ranges from 9.8% to 80%, and the variation in frequency depends on the adopted diagnostic criteria [2]. About 60–70% of the human population has at least one TMD symptom in their lifetime but only 5% of cases undertake the treatment [3]. With regard to gender, the literature reveals countless studies which indicating the occurrence of female predominance [4]. Women are twice as likely as men to suffer from TMD [5].

TMDs represents a group of musculoskeletal/neuromuscular conditions that may affect masticatory muscles, the temporomandibular joint (TMJ) and its associated structures [6,7]. The main symptoms and signs of TMD include masticatory pain, headaches, limited mandibular motion and its deviation pattern, TMJ noises, subluxation or/and jaw locked, jaw functional limitations, neck pain and poor sleep quality [8,9,10,11,12]. The etiology is multifactorial in nature and involves the overlapping of biological, behavioral, environmental, social, emotional and cognitive aspects [13,14]. These components can be considered as initiating, predisposing or perpetuating factors. The most common reasons concern direct and indirect trauma, repetitive microtrauma, systemic and local factors, postural and parafunctional habits, genetic determination as well as psychosocial findings relating to depression and anxiety [2,6,8,13,15,16,17,18,19]. The role of occlusion still remains unclear [20,21,22,23,24,25]. Causative factors can act independently, mutually and synergistically. In most cases it is usually impossible to identify a single leading factor.

TMDs reflects a group of comorbid conditions and clinical issues in which the incidence of muscular dysfunction tend to be more common than those which are intra-capsular joint-related [26,27,28]. Myofascial disturbances arise from tension, fatigue or spasm of the masticatory muscles whereas intra-articular disorders derive from mechanical or inflammatory damage to the joint itself. Muscle dysfunction seems to be the primary cause of TMD development [29]. 

Myofascial pain is one of the most common disorders of the temporomandibular joints, which represents a subtype of myalgia according to the first axis of the Diagnostic Criteria for Temporomandibular Disorders (DC/TMD) [1]. Some reports indicate that the overall prevalence of TMD myofascial pain amounts to 45.3% [30]. Other literature estimates that even more than 50% of temporomandibular disorders reflect myofascial pain [31]. As a temporomandibular disorder myofascial pain with referral is manifested through pain of muscle origin including pain spreading beyond the boundary of the masticatory muscles [32]. The limitation of mandibular motion occurs secondarily to pain [32]. Clinical pattern is always related to trigger points located in the head and neck region. As a consequence, autonomic, motor and sensory reactions appear, including local and transferred pain [30,33]. It should be noted that myofascial pain is the most common cause of orofacial pain, which modifies quality of life and biopsychosocial well-being [32,34,35]. Resultantly, depression, anxiety and somatic disorders appear. 

Currently, for better diagnosis and more efficient treatment of TMDs, instrumental analysis including electromyography, electrovibratography, electrosonography, electrognathography, thermography, electrokinetic and axiographic measurements, as well as the occlusal analysis system should be considered [27]. These biometric approaches enable quantitative assessment of the temporomandibular joint and bridge the gap between clinical procedures and TMJ Imaging [36].

The aim of this pilot study was to evaluate the temporomandibular joint, the prevalence of single and multiple diagnosis and potential sided domination of temporomandibular dysfunction in patients with temporomandibular disorder–myofascial pain with referral.

## 2. Materials and Methods

### 2.1. Ethical Issues

The study was approved by the Ethics Committee of the Medical University of Bialystok, Poland (permission number: R-I-002/322/2016). The research was carried out in accordance with the principles of the Declaration of Helsinki of the World Medical Association and the Guidelines for Good Clinical Practice. Participation in the study was voluntary. All the subjects obtained comprehensive information about the nature, scope of clinical activities and the course of the proceedings. At every stage, patients had the right to refuse to participate in the research without consequences. Participation in the study had been preceded by a written consent by every subject.

### 2.2. Subjects and the Size of the Sample

The study group enrolled 50 people—37 females and 13 males. All patients had complete natural dentition. They were referred to the Department of Prosthodontics at the Medical University of Bialystok, Poland. The study recruited people between 18 and 25 years old with an average age of 23.36 ± 2.14 years. All subjects underwent a clinical examination according to the Diagnostic Criteria for Temporomandibular Disorders (DC/TMD) (Axes I and II) and were identified with myofascial pain with referral (Axis I of DC/TMD) [37]. Qualification testing was conducted by a dentist who is also a physiotherapist (J.K.).

#### 2.2.1. Inclusion Criteria

- Myofascial pain with referral (Axis I of DC/TMD)
- Pain within the craniofacial and/or craniomandibular region (VAS, Visual Analogue Scale ≥ 8 points).
- Complete natural dentition (Class I of Angle’s Molar Classification, canine position).
- No history of orthodontic therapy or lack of retention status over 3 years after the treatment completion.

#### 2.2.2. Exclusion Criteria

- Trauma within the craniofacial and/or craniomandibular area.
- Surgical treatment within the craniofacial and/or craniomandibular region.
- Dental therapy supported by an occlusal splint.
- Prosthetic treatment and/or physiotherapy within the craniofacial and/or craniomandibular region in the medical history.
- Cases with possible health concerns affecting the function of the masticatory muscles.
- Metabolic diseases.
- Drugs.

The sample was described in detail in previous publications [5,33,38,39].

### 2.3. General Description of the Method

All patients underwent a thorough examination. The procedure followed:

- functional assessment of the masticatory system with respect to the Diagnostic Criteria for Temporomandibular Disorders (DC/TMD) [37]—axes I and II;
- Joint Vibration Analysis (JVA, BioResearch, Inc., Milwaukee, WI, USA);
- statistical analysis using the Statistica 13.1 (Statsoft Inc., Cracow, Poland), PQStat Software v. 1.8.2.182 (PQStat Software, Poznań, Poland) and G Power v. 3.1.9.4 (Germany).

### 2.4. General Description of the Joint Vibrations Analysis (BioJVA)

**Figure 1:**
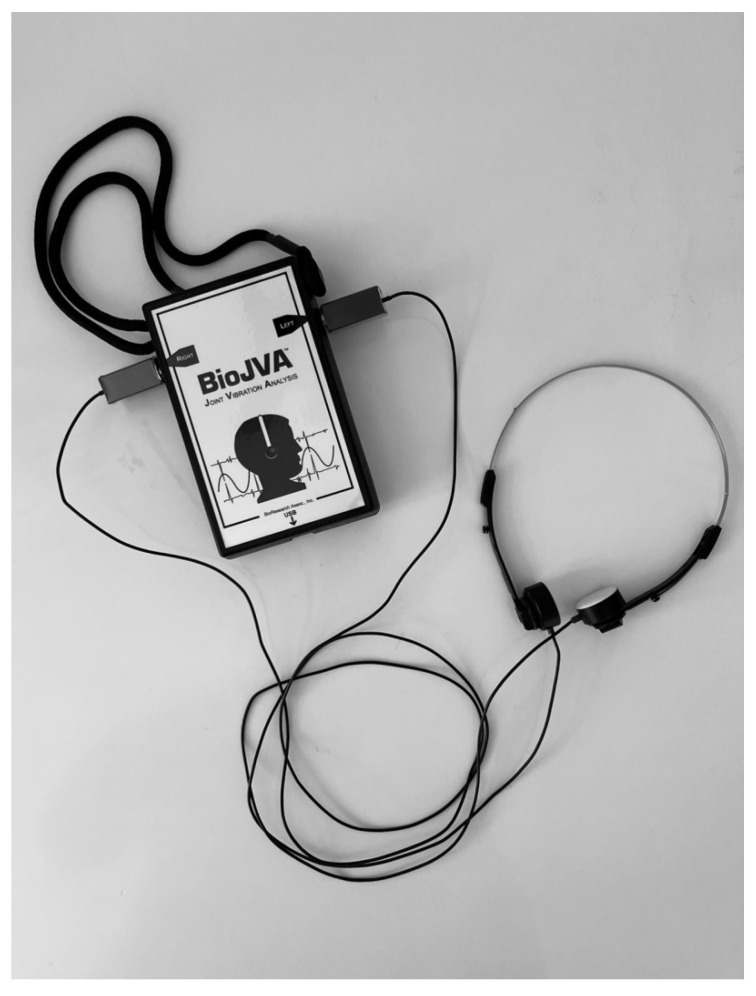
BioJVA Device (BioResearch, Inc., Milwaukee, WI, USA).

The BioJVA device enables bilateral registration of temporomandibular vibrations (Figure 1). Two preauricular sensors use the piezoelectric phenomenon. In order to suppress interference from the surrounding environment, the detectors are covered with a silicone mass ensuring plane contact with the skin of the person under examination. Temporomandibular joint vibrations generate pressure, which, by influencing the piezo crystals, releases an acoustic wave that is transferred directly onto the computer screen in real time and is expressed in graphs as dependence of vibration amplitude and time. The software (Biopak, BioResearch, Inc., Milwaukee, WI, USA) paired with the device allows for the specification each reported vibration.

The requirement for a reliable and correct single registration is repeatable translation of both condyles to their full extent—provided by a repeatable pre-declared range of the mouth opening—and the subsequent subtle intermaxillary occlusal contact during the clenching of both dental arches, coupled with the action of the metronome. Full condylar movements reveal limitations in the range of mandibular motion, deviations, deflections and potential temporomandibular vibrations. The subtle intermaxillary occlusal contacts facilitate verification of the maximum mandibular range of motion included in the single registration.

The range of motion (ROM) is specified as the distance between the incisal edges of the upper and lower central incisors in the position of maximum mouth opening enlarged by the overbite. Lateral deflection (LD) reflects the discrepancy between the interincisal point of the lower central incisors and the mid-sagittal plane in the position of the maximum mouth opening. Both measurements (ROM and LD) are expressed in millimeters and should be initially entered into the software at the beginning of the registrations [36].

The BioJVA allows for the classification of the condition of the individual temporomandibular joint in relation to Mark Piper’s classification, which was defined with respect to the results of Magnetic Resonance Imaging (MRI) and reflects modification of a classification scheme by Tasaki and Westesson [40,41]. The key stage of categorization is the analysis of the course of the vibration wave, taking into account the reference notation of the waveform (JVA Flow Chart) [36].

### 2.5. Joint Vibration Recording Procedure (BioJVA Procedure)

JVA registrations were performed in the morning, under the conditions of suggestive relaxation with no background noise, no side conversations and with no third parties accompanying the patient. A lack of visual, auditory and multisensory distractors (bright monitor light, radio and smartphones, respectively) was adhered to. The clinical procedure was performed by an experienced dentist specialized in prosthodontics and physiotherapist in one person (the author J.K).

**Figure 2:**
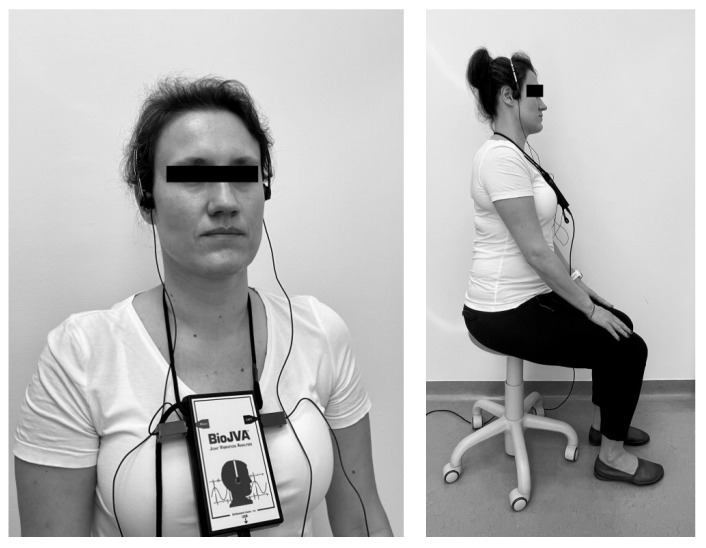
Position of the patient during the joint vibration recording procedure.

Two accelerometers mounted to the arch running over the cranial vault were positioned in the preauricular area directly over TMJ’s (Figure 2). Patients were asked to open the mouth as wide as possible, then clench both dental arches together to a subtle occlusal contact and repeat these activities several times (usually six or more complete cycles of the opening and closing of the mouth) following the metronome to control the velocity of the motion and the time of registration (10 s). One-minute breaks between consecutive registrations were respected. In order to reduce impedance, in each case the facial skin and accelerometers were rinsed with 2% salicylic alcohol.

During registration the patients remained upright and sitting in the initial default position of the adjustable office stool without lumbar support. Feet were positioned symmetrically on the floor, one foot apart from each other, hands rested on the thighs. The patients looked straight ahead. Prior to registration, the patients were oriented according to the Upright Posture Position of the Mandible (UPPM position) [42].

### 2.6. Statistical Analysis

Statistical analysis was performed using Statistica 13.1 (Statsoft Inc., Cracow, Poland), PQStat Software v. 1.8.2.182 (PQStat Software, Poznań, Poland) and G Power v. 3.1.9.4 (Germany). The arithmetic mean and median as well as measures of differentiation including standard deviation were calculated. To compare categorical variables Pearson’s chi-squared test of independence for a 2 × 2 contingency table was applied. A one-sided Fisher’s exact test was used in the case of small sample size when the expected number of frequencies was below 5. The Wilcoxon test was used to assess significant differences in the groups divided according to the side of the joint (right/left). 

Statistical significance was established at *p* < 0.05. For the one-sided Fisher’s exact test and Wilcoxon test, a post-hoc power analysis was conducted. The statistical power (1-β) was calculated as the function of the population effect size, sample size (*n*) and α. We additionally assessed the sample size required for the revealing of a statistically significant difference with respect to gender and to the side of the joint (right/left) at the 0.05 level with a probability of 0.8 (80%).

## 3. Results

Sixty seven percent (*n* = 67) of all examined temporomandibular joints were classified as group I, of which 33% (*n* = 33) were on the right side, and 34% (*n* = 34) were on the left side (Table 1). Class IIIA was showed in 17% (*n* = 17) joints and class IIIB in only 1% (*n* = 1). Eight percent (*n* = 8) of joints were classified as class IVA, 3% (*n* = 3) demonstrated class IVB. Two percent (*n* = 2) of joints were recorded in class VA and VB (Table 1). Sixty-eight percent (*n* = 50) of joints in females belonged to group I. In the case of males, the prevalence of this class was 65% (*n* = 17) (Table 1). Class IIIB, IVB and VB were not found in the group of males (Table 1). There were no statistically significant differences in the prevalence of temporomandibular disorder with respect to gender (Chi^2^ = 0.041467; *p* = 0.838639) (Table 1). The test’s power to detect the specified effect was on the low level (Fisher’s Exact Unilateral Test: *p* = 0.510052; (1-β) = 0.0570152) (Table 1). No sided domination in the occurrence of temporomandibular dysfunction was found (Table 1).

Sixty-two percent (*n* = 31) of the patients demonstrated a normal condition for both TMJs (Table 2). The bilateral stage 3A was reported in 10% of people. Four percent (*n* = 2) of the subjects showed the bilateral stage 4A. Two percent of patients suffered from the condition 4B. The remaining 22% of the patients suffered from different combinations of diagnoses for both temporomandibular joints (Table 2). There were no statistically significant differences in the prevalence of combined diagnosis with respect to gender. The test’s power to detect the specified effect was on a low level (Fisher’s Exact Unilateral Test: *p* = 0.60975; (1-β) = 0.0277777) (Table 2). No sided domination in the occurrence of temporomandibular dysfunction was found (Table 2).

In the entire study group, the mean of the total vibration energy (Total Integral) on the right side was 29.08 KPaHz and on the left was 31.88 KPaHz (Table 3). The median values were at the level of 7.20 and 7.85 KPaHz, respectively. In the case of vibration < 300 Hz, the results in the case of both joints were comparable and amounted to 25.05 KPaHz. There were no statistically significant differences of the Total Integral and Integral < 300 Hz between the right and left temporomandibular joints (*p* > 0.05) (Table 3). The total energy of vibrations for frequencies > 300 Hz were 4.04 KPaHz on the right and 6.81 KPaHz on the left side. 

The mean peak frequency (Peak FreQ) was 48.74 Hz within the right TMJ and 71.52 Hz in the left one. The vibration frequency (Med. FreQ) amounted to 111.88 Hz on the right, while on the left it was 148.14 Hz (Table 3). Statistically significant differences of the Integral > 300 Hz, > 300/< 300 Hz Ratio, Peak FreQ and Med. FreQ were noted between the right and left temporomandibular joints (*p* < 0.05). Higher values of these parameters were reported on the left side (Table 3). The maximum range of mouth opening within the entire study group was 49.90 mm (Table 3).

Within the female group, the mean total energy of vibrations on the right side was 32.89 KPaHz and on the left was 31.86 KPaHz (Table 4). The median values were 8.00 and 8.20 KPaHz, respectively. In the case of vibrations < 300 KPaHz, the results were at the level of 28.87 KPaHz on the right side and 25.14 KPaHz on the left side. The total vibration energy for frequencies > 300 Hz on the right amounted to 4.03 KPaHz and on the left was 6.73 KPaHz. 

The mean peak frequency was 43.68 Hz on the right and 75.11 Hz on the left. The vibration frequency (Med. FreQ) amounted to 114.05 Hz within the right joint and on the left was 149.11 Hz. Statistically significant differences of the >300/<300 Hz Ratio, Peak Frequency and Median Frequency were noted between the right and left temporomandibular joints (*p* < 0.05). Higher values of these parameters were reported on the left side (Table 4). The maximum range of mouth opening was 48.95 mm (Table 4).

In the case of males, the mean total vibration energy on the right was 18.24 KPaHz and on the left was 31.94 KPaHz. The median values amounted to the level of 6.40 KPaHz and 6.90 KPaHz, respectively. In the case of vibrations <300 Hz, the results reached 14.16 KPaHz on the right side and 24.82 KPaHz on the left side. The total energy of vibrations for frequencies > 300 Hz on the right side was 4.07 KpaHz and on the left was 7.06 KPaHz. 

The mean peak frequency was 63.15 Hz on the right and 61.31 Hz on the left. The vibration frequency (Med. FreQ) amounted to 105.69 Hz on the right side, while on the left it was 145.38 Hz. Statistically significant differences between right and left temporomandibular joints were noted only in the case of the Median Frequency (*p* < 0.05). A higher value for this parameter was reported on the left side (Table 5). The maximum range of mouth opening for the male group was 52.62 mm (Table 5).

## 4. Discussion

The prevalence of temporomandibular disorders shows variation and depends on the population studied and profile of the conducted research. The presented study revealed that many patients had more than one physical diagnosis of TMD (Table 1 and Table 2). In 28% of people, a combination of myofascial pain with referral and bilateral temporomandibular dysfunction was noted (Table 2). Ten percent of the patients revealed a dual diagnosis including myofascial pain with referral and disorder of one of the temporomandibular joints (Table 2). 

These observations remain in line with previous findings. John et al. observed that, among 416 females, 266 received one diagnosis, 117 had two diagnoses, 32 subjects had three diagnoses and 1 person obtained four diagnoses. These authors emphasized that such condition is possible because the eight diagnostic categories of Research Diagnostic Criteria for Temporomandibular Disorders (RDC/TMD) are not mutually exclusive [43]. With respect to DC/TMD, Więckiewicz et al. revealed that 55.9% of 213 individuals demonstrated pain-related TMD including myalgia, myofascial pain, arthralgia and headache attributed to TMD, and 48.8% reported temporomandibular disorders, mainly disc displacement with reduction (47.4%). Furthermore, a total of 73% of cases had suffered from headaches in the previous 12 months [44].

According to RDC/TMD, Osiewicz et al. revealed that among the Polish patient population, 38% of the subjects demonstrated the condition of multiple diagnoses, which was defined as a combination of the muscle disorders (I), disc displacement (II) and arthralgia, osteoarthritis and/or osteoarthrosis (III) [45]. A combination of myofascial pain with or without limited opening and disc displacement was observed in 20.5% of cases. Myofascial pain with or without limited opening and arthralgia was noted in 8.6% of the patients [45]. 

A triple diagnosis was observed as a combination of myofascial pain with or without limited opening with osteoarthritis and osteoarthrosis and myofascial pain with or without limited opening with disc displacement and arthralgia. The prevalence of the above mentioned combined conditions was 3.3% and 1.3%, respectively. Osiewicz et al. highlighted that in future investigations, a lower incidence of myofascial pain could be expected [45]. The reason for that is the fact that currently used DC/TMD protocol are much more restrictive than previously used RDC/TMD where it concerns the criteria for the diagnosis of myofascial pain [45].

With respect to DC/TMD and selected Northern Jordanian population aged between 18 and 78 years, the frequency of myofascial pain with referral amounted to 2.2% [28]. A combination of myofascial pain with referral and arthralgia was reported at a similar level in 2.2% of cases. Myofascial pain without a referral pattern and arthralgia was noted in 0.8% of the patients. Local myalgia and arthralgia was reported in 0.3% of the subjects [28]. According to RDC/TMD, Rauch et al. stated that, among the German population, 34.4% of people received no TMD diagnosis. The next 65.6% suffered from TMD [46]. Of those patients 55.5% obtained single, and 44.5% received multiple diagnoses [46].

According to the diagnostic criteria of the American Academy of Orofacial Pain (AAOP), Machado et al. revealed that only 6.7% of the patients had only one diagnosis [47]. In 93.3% of the cases, patients had at least one more problem diagnosed along with the main diagnosis. In 13.7% of people, five different conditions were simultaneously recognized. As the main diagnosis, masticatory myofascial pain was reported in 10.4% of the patients [47]. As the complex of the main and additional diagnoses, masticatory myofascial pain was demonstrated in 24.1% of people.

The presence of multiple diagnoses broadens the area for treatment possibilities. In addition to causal treatment aimed at treating multi-dimensional myofascial pain with referral, it is necessary to take steps to alleviate intra-articular dysfunction. The most accepted model considers directed and self-directed biopsychosocial profile modulation and symptomatic treatment [30]. 

The first solution includes a proposal of cognitive behavioural therapy, biofeedback, sleep hygiene measures and relaxation techniques [30]. Symptomatic treatment involves occlusal splints, pharmacological therapy, trigger point therapy, soft tissue mobilization, occlusal equilibration, prosthodontic reconstruction, acupuncture, botulinum toxin, transcutaneous electrical neuromuscular stimulation (TENS) and/or contingent electrical stimulation [30,38]. The new challenge in TMD management entails the creation of a relevant set of biomarkers for temporomandibular disorders—quantitative sensory measures and a genomic or molecular profile [1].

In presented study, only 33% (*n* = 33) of all temporomandibular joints (*n* = 100) were affected by the pathology (Table 1). However, the presence of even these structural and/or functional changes may indicate profound progression in temporomandibular dysfunction. As previously mentioned, the prevalence of muscular disorder tend to be more common than those that are intracapsular joint-related [26,27,28] and musculoskeletal dysfunction appear to be the primary source of TMD development [29].

Machado et al. highlighted that patients with chronic muscle dysfunction exposed to long-term parafunctional habits—especially clenching—revealed secondary organic changes within TMJ [47]. In turn, in the case of primary TMJ pathology, subsequent muscle symptoms could appear as a consequence of the protective splinting of the jaws. Through deep pain and by creating cyclical muscle pain, this last mechanism may lead to the incidence of cumulative trauma [47]. It should be highlighted that triggers for myofascial pain include emotional stress, tension, fatigue, overloads, nutritional deficiencies, infections, unhealthy behavior and poor ergonomics [38]. 

It is also well known that changes in head posture influence a response within the masticatory system including the biomechanical behavior of TMJ and its associated structures [48,49]. Head position affects the resting position of the mandible, modifies muscular activity and alters the internal arrangement of the TMJ. A close link exists between head and cervical posture improvement and relief of the symptoms of temporomandibular joint [48]. 

The above mentioned feedback could be reflected by the anatomophysiological components of C0–C2 complex (Occiput-Axis) and convergence phenomenon within cervical and trigeminal afferents in the trigeminocervical nucleus [5,50]. In summary, in temporomandibular disorder, researchers should pay attention to the cervical spine and associated head position, the phenomenon of convergence and the central sensitization of pain [51,52,53].

In the presented study, 31 patients demonstrated a healthy condition of both temporomandibular joints (Table 2). Bearing in mind the main diagnosis—myofascial pain with referral—this may support the suggestion that muscle dysfunction precedes joint disorders and that muscle dysfunction appears as the primary cause of TMD development. This suggestion seems to be in line with Wolff’s law and bone functional adaptation as well as the biotensegrity model of TMJ [33,54]. To summarize, “form follows function” [22]. On the other hand, early detection of muscle dysfunction enables the implementation of appropriate primary, secondary or tertiary prevention [55,56].

With respect to JVA, the estimation of the Total Integral enables division of the spectrum of TMJ vibrations into four groups of intensity—small (0–20 KPaHZ), medium (20–80 KPaHZ), large (80–300 KPaHZ) and very large vibrations (>300 KPaHZ) (Table 3, Table 4 and Table 5) [36]. Very large vibrations may indicate the acute phase of disc displacement with reduction (stage Piper 4A). When the acute phase becomes more chronic, the Total Integral decreases [36]. Radke et al. emphasized that, in the case of acute or the well adapted stage of Piper 4B, a low level of Total Integral could be observed. During adaptation of the Piper 4B condition, medium results were reported [36].

The results of the JVA correspond with other previous findings. Kondrat et al. showed that, in a group of healthy people (*n* = 186) with an average age of 19 years, the Total Integral amounted to 39.02 ± 63.97 KPaHz among females (Me = 16.35 KPaHz) and to 39.02 ± 67.92 KPaHz in males (Me = 19.45 KPaHz) [41]. In our study, this variable scored 32.37 ± 66.39 KPaHz (Me = 8.10 KPaHz) in females and 25.09 ± 41.72 KPaHz (Me = 6.65 KPaHz) in males (Table 4 and Table 5). Slight differences between both study results are likely conditioned by the different sample sizes and prevalence of people with healthy joints within the entire study group.

Kondrat et al. revealed that Integral < 300 Hz amounted to 31.33 ± 54.83 KPaHz (Me = 14.15 KPaHz) in females and 35.86 ± 63.41 KPaHz (Me = 18.05 KPaHz) in males [41]. In our study, this outcome reached 27.00 ± 56.05 KpaHz (Me = 7.10 KpaHz) in females and 19.49 ± 32.46 (Me = 5.80 KpaHz) in males (Table 4 and Table 5). Integral < 300 Hz is associated with incorrect disc movements [36]. Values of this outcome close to the Total Integral prove that the reported vibration results from irregular disc motion, not from any other degenerative changes. On the other hand, as the main vibration, the Integral >300 Hz reflects relative roughness of the sliding surfaces within TMJ and is considered only in the case of small and medium amplitude vibrations [36].

Kondrat et al. reported that the Ratio > 300/< 300 Hz was 0.15 ± 0.15 in females and 0.14 ± 0.14 in males [41]. In our study, this outcome amounted to 0.23 ± 0.17 in females and 0.26 ± 0.15 in males (Table 4 and Table 5). Evaluation of the Ratio > 300/< 300 Hz permits avoidance of overestimating any degenerative changes with large or very large vibrations. This outcome is not considered in the case of small vibrations. The reason is the potential background of electrical noises that represent artifacts [36].

The Peak Amplitude is taken into account in the case of hearing noise within TMJs that are reported by the patients. Values below 6.0 (Newtons/meter^2^; Pa) appear to be a crepitus. Results above 6.0 Pa are typical in normal hearing [36].

The next outcome described as a Peak Frequency indicates long-term chronicity. Long-lasting temporomandibular pathology means lower values of Peak Frequency. Similar to the Peak Frequency, the Median Frequency suggests chronicity of TMD. In the case of a discrepancy between values of the Median Frequency and the Peak Frequency and by the dominance of the Median Frequency, there exists some possibility of active degenerative condition occurrence [36].

Despite the fact that there was no sided domination (left/right) in the prevalence of temporomandibular disorder (Table 1), all statistically significant differences reported with respect to JVA suggest profound progression of dysfunction within the left TMJ compared to the right one (Table 3, Table 4 and Table 5). This lateralization could reflect descending or ascending cranio-mandibular, homo- or heterolateral dysfunction [57].

### Strengths and Limitations of the Study

This is probably the first study on joint vibration analysis performed in patients with temporomandibular joint disorders – myofascial pain with referral – who were diagnosed with respect to DC/TMD. Applied protocol allowed for the selection of a homogenous group of patients with regard to the strictly defined research criteria. 

The main advantage is the fact that JVA is a reliable screening tool with a specificity of 98% [36]. As opposed to palpation and auscultation with stethoscope JVA enables timely, quantitative estimation of dynamic function of the temporomandibular joints and evaluation of the degree of temporomandibular sounds [36]. It supports clinical diagnosis and indicates the need to perform CBCT or MRI [36]. 

The main limitation of the study was the small sample size and the possibility of bias regarding selection, measurement and confounding factors. Thus, the presented data should be treated with caution. Selection bias usually arises from the general study design or/and data collection. The current research favors details from patients with temporomandibular joint disorder including myofascial pain with referral, who were additionally selected with respect to the strictly defined inclusion and exclusion criteria. In an unbiased sample, differences observed between the cases of units from a population and those from the entire population they represent, should originate only by chance. Otherwise, it would indicate selection bias. 

Another issue is measurement bias. As mentioned before, BioJVA is a reliable device with very high sensitivity. However, proper registration requires repeatable, individually declared mouth opening. Sometimes, in the case of patients with acute pain this may be difficult to achieve and may result in omission of important vibrations. Also in the case of inflammatory exudate that separates the anatomical structures in the temporomandibular joint, the perception of vibrations may be reduced. Head position and posture during registration also could play a significant role.

Confounding bias refers to an inappropriate association made between an outcome and a factor. It suggests some relationship that does not exist or masks a true relationship. Due to the specificity of the presented study group and the tested differences, the following confounding factors should be considered: sample size, age, gender, one sided or bilateral chewing, individual morphology of temporomandibular joints, posture disorders and lateralization.

## 5. Conclusions

The relatively high prevalence of multiple diagnoses proved the overlapping nature of muscle and intraarticular disorders. Twenty eight percent of the subjects suffered from a combination of myofascial pain with referral and bilateral temporomandibular dysfunction. In 62% of the patients, a lack of intraarticular disorders was reported. The suggestion that there exists sided domination in the occurrence of temporomandibular disorders has not been confirmed. Due to the small sample size, such differences cannot be excluded. With regard to other limitations of the study, further research is needed.

## Figures and Tables

**Table 1:** The prevalence of temporomandibular disorder with respect to M. Piper’s classification model in the entire group (*n* = 50), the female group (*n* = 37) and the male group (*n* = 13).

M. Piper’s Classification										Comparison with Respect to Gender					
	Entire Group *n* = 50			Female Group *n* = 37			Male Group *n* = 13			All Joints in Group I vs. All Joints in Group II, IIIa, IIIb, IVa, IVb, Va and Vb					
	All Joints	Right Joints	Left Joints	All Joints	Right Joints	Left Joints	All Joints	Right Joints	Left Joints	Chi^2^ Pearsona			Fisher’s Exact Unilateral Test		
	*n* = 100	*n* = 50	*n* = 50	*n* = 74	*n* = 37	*n* = 37	*n* = 26	*n* = 13	*n* = 13	Chi^2^	df	*p*-Value	*p*-Value	Power (1-β)	Sample Size for 80% Test Power (n)
I	67 (67%)	33 (66%)	34 (68%)	50 (68%)	25 (68%)	25 (68%)	17 (65%)	8 (62%)	9 (69%)	0.041467	1	0.838639	0.510052	0.0570152	8110
II	0 (0%)	0 (0%)	0 (0%)	0 (0%)	0 (0%)	0 (0%)	0 (0%)	0 (0%)	0 (0%)						
IIIA	17 (17%)	8 (16%)	9 (18%)	10 (14%)	4 (11%)	6 (16%)	7 (27%)	4 (31%)	3 (23%)						
IIIB	1 (1%)	1 (2%)	0 (0%)	1 (1%)	1 (3%)	0 (0%)	0 (0%)	0 (0%)	0 (0%)						
IVA	8 (8%)	3 (6%)	5 (10%)	7 (9%)	3 (8%)	4 (11%)	1 (4%)	0 (0%)	1 (8%)						
IVB	3 (3%)	2 (4%)	1 (2%)	3 (4%0	2 (5%)	1 (3%)	0 (0%)	0 (0%)	0 (0%)						
VA	2 (2%)	1 (2%)	1 (2%)	1 (1%)	0 (0%)	1 (3%)	1 (4%)	1 (8%)	0 (0%)						
VB	2 (%)	2 (4%)	0 (0%)	2 (3%)	2 (5%)	0 (0%)	0 (0%)	0 (0%)	0 (0%)						

% percentage within column (% all joints; % right joints; % left joints). M. Piper’s classification of TMJ damage [40,41] I—Normal; II—Ligaments or cartilage damage; IIIA—Partial disc subluxation, with reduction; IIIB—Partial disc subluxation, non-reducing; IVA—Complete disc dislocation, with reduction; IVB—Complete disc dislocation, non-reducing; VA—No disc, Bone to bone—Adapting; VB—No disc and Bone to bone—Adapted.

**Table 2:** The prevalence of combined bilateral diagnosis of temporomandibular dysfunction with respect to M. Piper’s classification model in the entire group (*n =* 100), the female group (*n* = 74) and the male group (*n* = 26).

M. Piper’s Classification				Comparison with Respect to Gender		
				I—Both TMJ’s vs. All Other Combination		
	Entire Group	Female Group	Male Group	Fisher’s Exact Unilateral Test		
	*n* = 50	*n* = 37	*n* = 13	*p*-Value	Power (1-β)	Sample Size for 80% Test Power (n)
I—both TMJ’s	31 (62%)	23(62.16%)	8(61.54%)	0.60975	0.0277777	197,990
3A—both TMJ’s	5(10%)	2(5.41%)	3(23.08%)			
4A—both TMJ’s	2(4%)	2(5.41%)	0(0.00%)			
4B—both TMJ’s	1(2%)	1(2.70%)	0(0.00%)			
I—left TMJ, 3A—right TMJ	2 (4%)	1 (2.70%)	1 (7.69%)			
I—left TMJ, 3B—right TMJ	1(2%)	1(2.70%)	0(0.00%)			
3A—left TMJ, I—right TMJ	1(2%)	1(2.70%)	0(0.00%)			
3A—left TMJ, 4A—right TMJ	1(2%)	1(2.70%)	0(0.00%)			
3A—left TMJ, 5B—right TMJ	2(4%)	2(5.41%)	0(0.00%)			
4A—left TMJ, I—right TMJ	1 (2%)	1(2.70%)	0(0.00%)			
4A—left TMJ, 3A—right TMJ	1 (2%)	1(2.70%)	0(0.00%)			
4A—left TMJ, 5A- right TMJ	1(2%)	0(0.00%)	1(7.69%)			
5A—left TMJ, 4B—right TMJ	1 (2%)	1(2.70%)	0(0.00%)			

% percentage within column (% all joints; % right joints; % left joints). M. Piper’s classification of TMJ damage [40,41]: I—Normal; II—Ligaments or cartilage damage; IIIA—Partial disc subluxation, with reduction; IIIB—Partial disc subluxation, non-reducing; IVA—Complete disc dislocation, with reduction; IVB—Complete disc dislocation, non-reducing; VA—No disc, Bone to bone—Adapting; VB—No disc and Bone to bone—Adapted.

**Table 3:** Parameters of temporomandibular joint vibration analysis in the entire study group (*n* = 50). The mean values, standard deviation (±SD), median (Me) and *p*-value are given.

Parameters of Joint Vibration Analysis	All Joints in the Entire Study Group *n* = 100			All Right Joints in the Entire Study Group *n* = 50			All Left Joints in the Entire Study Group *n* = 50			Comparison between Right and Left Joints		
										Wilcoxon Test		
	Mean	±SD	Me	Mean	±SD	Me	Mean	±SD	Me	*p*-Value	Power (1-β)	Sample Size for 80% Test Power (n)
Total Integral	30.48	60.83	7.75	29.08	65.53	7.20	31.88	56.37	7.85	0.699400	0.0910029	3125
Integral < 300 Hz	25.05	50.92	6.45	25.05	58.23	6.50	25.05	43.00	6.45	-	-	-
Integral > 300 Hz	5.43	12.47	1.00	4.04	8.21	0.80	6.81	15.59	1.25	0.033018 *	0.4019170	160
>300/<300 Ratio	0.24	0.16	0.22	0.21	0.13	0.19	0.27	0.18	0.24	0.002664 *	0.8142656	49
Peak Amplitude	2.00	3.79	0.70	2.18	4.63	0.90	1.81	2.74	0.65	0.100122	0.1538804	771
Peak FreQ	60.13	59.62	37.00	48.74	50.95	25.00	71.52	65.74	56.00	0.003856 *	0.8294044	46
Med. FreQ	130.01	54.10	128.00	111.88	50.80	103.00	148.14	51.59	140.00	0.000001 *	0.9992546	15
Max Opening	49.90	5.69	50.50	49.90	5.69	50.50	49.90	5.69	50.50	-	-	-

* *p* < 0.05 statistical significance; Vibration frequency: Small vibrations—Total Integral = 0–20 Hz; Medium vibrations—Total Integral = 20–80 Hz; Large vibrations—Total Integral = 80–300 Hz; IV Very large vibrations = Total Integral ≥ 300 Hz; Primary outcome: Total integral—defined as a total of the pressure waves over time given in KPaHz; the area under the mean Fast Fourier Transform (FFT) frequency distribution of all marked vibrations; this variable allows for the division of the spectrum of TMJ vibrations into four groups (small, medium, large and very large) with respect to the JVA Flow Chart Range of motion (Max. Opening)—the distance between incisal edges of the upper and lower central incisors in the position of maximum mouth opening; expressed in mm; Integral >300 Hz—this is a total integral component covering frequencies above 300 Hz, reflecting rough surfaces, expressed in KPaHz; Ratio >300 Hz < 300 Hz—this mirrors the ratio of two integrals of the two different frequency ranges above and below 300 Hz; Secondary outcome: Integral < 300 Hz—this is a total integral component covering frequencies below 300 Hz, reflecting disk movements, expressed in KpaHz; Peak Amplitude—this mirrors the mean intensity of the Peak Frequency, expressed in Pa; Peak Frequency—defined as the frequency with the highest amplitude of all the measured frequencies, expressed in Hz; Median Frequency—specified as the frequency in the middle of the entire frequency range such that half of the total energy is above and half below, expressed in Hz [36].

**Table 4:** Parameters of temporomandibular joint vibration analysis in the female group (*n* = 37). The mean values, standard deviation (±SD), median (Me) and *p*-value are given.

Parameters of Joint Vibration Analysis	All joints in the Entire Study Group *n* = 74			All Right Joints in the Entire Study Group *n* = 37			All Left Joints in the Entire Study Group *n* = 37			Comparison between Right and Left Joints		
										Wilcoxon Test		
	Mean	±SD	Me	Mean	±SD	Me	Mean	±SD	Me	*p*-Value	Power (1-β)	Sample Size for 80% Test Power (n)
Total Integral	32.37	66.39	8.10	32.89	75.09	8.00	31.86	57.44	8.20	0.700460	0.0597848	28,225
Integral < 300 Hz	27.00	56.05	7.10	28.87	66.84	7.20	25.14	43.54	7.00	0.832725	0.1011661	1609
Integral > 300 Hz	5.38	13.42	1.10	4.03	9.06	0.90	6.73	16.71	1.30	0.101808	0.2880997	188
>300/<300 Ratio	0.23	0.17	0.22	0.20	0.12	0.14	0.27	0.20	0.23	0.008289 *	0.7561951	42
Peak Amplitude	2.20	4.28	0.70	2.48	5.33	0.90	1.92	2.93	0.70	0.234751	0.1738566	443
Peak FreQ	59.39	60.47	37.00	43.68	41.95	25.00	75.11	71.74	56.00	0.004789 *	0.9011432	28
Med. FreQ	131.58	51.94	130.00	114.05	43.92	11500	149.11	53.95	142.00	0.000020 *	0.9931255	15
Max Opening	48.95	5.13	49.00	48.95	5.13	49.00	48.95	5.13	49.00	-	-	-

* *p* < 0.05 statistical significance; Vibration frequency: Small vibrations—Total Integral = 0–20 Hz; Medium vibrations—Total Integral = 20–80 Hz; Large vibrations—Total Integral = 80–300 Hz; IV Very large vibrations = Total Integral ≥ 300 Hz; Primary outcome: Total integral—defined as a total of the pressure waves over time given in KPaHz; the area under the mean Fast Fourier Transform (FFT) frequency distribution of all marked vibrations; this variable allows for the division of the spectrum of TMJ vibrations into four groups (small, medium, large and very large) with respect to the JVA Flow Chart Range of motion (Max. Opening)—the distance between incisal edges of the upper and lower central incisors in the position of maximum mouth opening; expressed in mm; Integral >300 Hz—this is a total integral component covering frequencies above 300 Hz, reflecting rough surfaces, expressed in KPaHz; Ratio >300 Hz < 300 Hz—this mirrors the ratio of two integrals of the two different frequency ranges above and below 300 Hz; Secondary outcome: Integral < 300 Hz—this is a total integral component covering frequencies below 300 Hz, reflecting disk movements, expressed in KpaHz; Peak Amplitude—this mirrors the mean intensity of the Peak Frequency, expressed in Pa; Peak Frequency—defined as the frequency with the highest amplitude of all the measured frequencies, expressed in Hz; Median Frequency—specified as the frequency in the middle of the entire frequency range such that half of the total energy is above and half below, expressed in Hz [36].

**Table 5:** Parameters of temporomandibular joint vibration analysis in the male group (*n* = 13). The mean values, standard deviation (± SD), median (Me) and *p*-value are given.

Parameters of Joint Vibration Analysis	All Joints in the Entire Study Group *n* = 26			All Right Joints in the Entire Study Group *n* = 13			All Left Joints in the Entire Study Group *n* = 13			Comparison between Right and Left Joints		
										Wilcoxon Test		
	Mean	±SD	Me	Mean	±SD	Me	Mean	±SD	Me	*p*-Value	Power (1-β)	Sample Size for 80% Test Power (n)
Total Integral	25.09	41.72	6.65	18.24	21.14	6.40	31.94	55.47	6.90	0.944285	0.2394969	83
Integral < 300 Hz	19.49	32.46	5.80	14.16	16.46	5.70	24.82	43.16	5.90	0.506746	0.2395178	83
Integral > 300 Hz	5.57	9.51	0.75	4.07	5.40	0.70	7.06	12.42	0.90	0.139415	0.2340410	86
>300/<300 Ratio	0.26	0.15	0.22	0.24	0.17	0.22	0.28	0.12	0.26	0.182339	0.2211755	95
Peak Amplitude	1.42	1.69	0.90	1.35	1.05	1.00	1.49	2.21	0.60	0.278708	0.0804391	1213
Peak FreQ	62.23	58.26	35.00	63.15	70.82	29.00	61.31	45.33	41.00	0.366986	0.0609899	7383
Med. FreQ	125.54	60.70	111.00	105.69	68.52	91.00	145.38	46.12	138.00	0.023130 *	0.7005520	17
Max Opening	52.62	6.53	54.00	52.62	6.53	54.00	52.62	6.53	54.00	-	-	-

* *p* < 0.05 statistical significance; Vibration frequency: Small vibrations—Total Integral = 0–20 Hz; Medium vibrations—Total Integral = 20–80 Hz; Large vibrations—Total Integral = 80–300 Hz; IV Very large vibrations = Total Integral ≥ 300 Hz; Primary outcome: Total integral—defined as a total of the pressure waves over time given in KPaHz; the area under the mean Fast Fourier Transform (FFT) frequency distribution of all marked vibrations; this variable allows for the division of the spectrum of TMJ vibrations into four groups (small, medium, large and very large) with respect to the JVA Flow Chart Range of motion (Max. Opening)—the distance between incisal edges of the upper and lower central incisors in the position of maximum mouth opening; expressed in mm; Integral >300 Hz—this is a total integral component covering frequencies above 300 Hz, reflecting rough surfaces, expressed in KPaHz; Ratio >300 Hz < 300 Hz—this mirrors the ratio of two integrals of the two different frequency ranges above and below 300 Hz; Secondary outcome: Integral < 300 Hz—this is a total integral component covering frequencies below 300 Hz, reflecting disk movements, expressed in KpaHz; Peak Amplitude—this mirrors the mean intensity of the Peak Frequency, expressed in Pa; Peak Frequency—defined as the frequency with the highest amplitude of all the measured frequencies, expressed in Hz; Median Frequency—specified as the frequency in the middle of the entire frequency range such that half of the total energy is above and half below, expressed in Hz [36].

## Data Availability

The article contains complete data used to support the findings of this study.
